# Prevalence of mastitis in dairy animals in Indonesia: A systematic review and meta-analysis

**DOI:** 10.14202/vetworld.2023.1380-1389

**Published:** 2023-07-04

**Authors:** Dian Meididewi Nuraini, Morsid Andityas, Peerapol Sukon, Patchara Phuektes

**Affiliations:** 1 Veterinary Science Program, Faculty of Veterinary Medicine, Khon Kaen University, Khon Kaen, Thailand; 2 Department of Animal Science, Faculty of Animal Science, Universitas Sebelas Maret, Surakarta, Indonesia; 3 Veterinary Technology Study Program, Department of Bioresources Technology and Veterinary, Vocational College, Universitas Gadjah Mada, Yogyakarta, Indonesia; 4 Research Program on Toxic Substances, Microorganisms and Feed Additives in Livestock and Aquatic Animals for Food Safety, Khon Kaen University, Khon Kaen, Thailand

**Keywords:** animals, cow, Indonesia, subclinical mastitis

## Abstract

**Background and Aim:**

Mastitis is an important disease that can reduce milk production and farmer income as well as negatively affect human health. This study aimed to summarize dairy mastitis in Indonesia, both subclinical mastitis (SCM) and clinical mastitis (CM), and its prevalence in different provinces, the diagnostic methods, and the animal species.

**Materials and Methods:**

Relevant studies on mastitis in dairy animals in Indonesia were obtained from PubMed, Scopus, ProQuest, Google Scholar, and Garuda. The title and abstract were screened for the eligibility of the studies. The full text of the selected studies was assessed and the data were extracted for analysis. To determine the pooled estimate of the prevalence of mastitis, a random-effects model was performed using the “Meta” and “Metaphor” packages in the R software version 4.2.2. The heterogeneity of several characteristics (mastitis type, provinces, animal species, and diagnostic methods) was evaluated through subgroup meta-analysis. Meta-regression analysis was conducted to assess the trend of mastitis prevalence reports over time. Publication bias was evaluated using Egger’s test and a funnel plot.

**Results:**

A total of 735 studies were retrieved for the title and abstract screening, which resulted in the final selection of 37 studies with a total of 6050 samples for meta-analysis. The pooled estimate of mastitis prevalence in dairy animals in Indonesia was 59.44% (95% confidence interval [CI], 52.39%–66.49%). Based on mastitis type, SCM had a significantly higher prevalence than CM (58.24% [95% CI, 51.26%–65.23%] vs. 3.31% [95% CI, 1.42%–5.19%]). No significant difference was observed in the analysis of other subgroups. Among provinces, Central Java had the highest prevalence (66.62% [95% CI, 49.37%–83.87%]), whereas Yogyakarta had the lowest (41.77% [95% CI, 14.96%–68.58%]). Based on animal species, cow and goat had a prevalence of 63.42% (95% CI, 55.97%–70.86%) and 44.96% (95% CI, 28.26%–61.66%), respectively. Based on the diagnostic method, the California mastitis test resulted in 60.08% (95% CI, 52.11%–68.06%) and the Institut Pertanian Bogor test, 56.00% (95% CI, 41.20%–70.81%). No significant change in the prevalence of mastitis in Indonesia was observed from 2003 to 2022.

**Conclusion:**

This study demonstrates that the pooled estimate of mastitis prevalence in dairy animals in Indonesia is >50%. Based on subgroup analysis, SCM had a higher prevalence than CM; however, the prevalence between provinces, detection methods, and animal species in the 2003–2022 periods was not significantly different. A mastitis control strategy needs to be developed to reduce the prevalence of mastitis and further loss in milk production.

## Introduction

In developing countries such as Indonesia, mastitis is one of the most prevalent and costly diseases in the dairy industry; it reduces milk production, alters milk quality, and eventually reduces farmers’ income [1, 2]. Mastitis is an infection of the mammary gland. It is characterized by high somatic cell count (SCC) in milk. Subclinical mastitis (SCM) is mainly characterized by high SCC. In contrast, the signs of clinical mastitis (CM) are redness, swelling, pain, and heat, often followed by abnormal milk appearance (i.e., clot, blood) [3]. Due to its visible clinical signs, CM could be easily detected by farmers and veterinarians, making it relatively easy to treat. Subclinical mastitis, on the other hand, has no visible clinical signs and can only be detected through indirect assessment or laboratory tests. Thus, it is difficult to diagnose and is thus suggested to have a higher prevalence and cause higher economic loss than CM [3].

The milk production in Indonesia cannot fulfill the national demand. It provides only approximately 22.7% of the national consumption, causing Indonesia to rely on >75% of imported milk [4]. On the other hand, the Indonesian population keeps increasing throughout the year, which means the demand for milk also increases. It highlights the importance of improving the dairy industry. However, mastitis has become a problem in increasing milk production. In addition to decreased milk production, several microorganisms and somatic cells present in mastitis milk could alter milk quality [5]. Furthermore, milk and milk products may transmit these microorganisms to humans. Consuming unpasteurized milk could lead to the transmission of illness caused by *Staphylococcus* spp., *Escherichia coli*, *Campylobacter*, *Yersinia*, and *Salmonella* [6]. Thus, mastitis as a concern in the dairy industry should be addressed.

Sudarwanto *et al*. [7] reported 83% of mastitis prevalence in dairy cattle in Indonesia. However, to the best of our knowledge, there are no systematic reviews and meta-analyses regarding this issue. A systematic review and meta-analysis can provide data on the pooled mastitis prevalence and subgroup prevalence estimates. Thus, this study aimed to summarizes mastitis, both SCM and CM, and its prevalence in dairy animals in Indonesia based on provinces, diagnostic methods, and animal species.

## Materials and Methods

### Ethical approval

This study used data from previous reports. No animal or animal product was used in this study, so ethical clearance was not required.

### Study period and location

**Figure-1:**
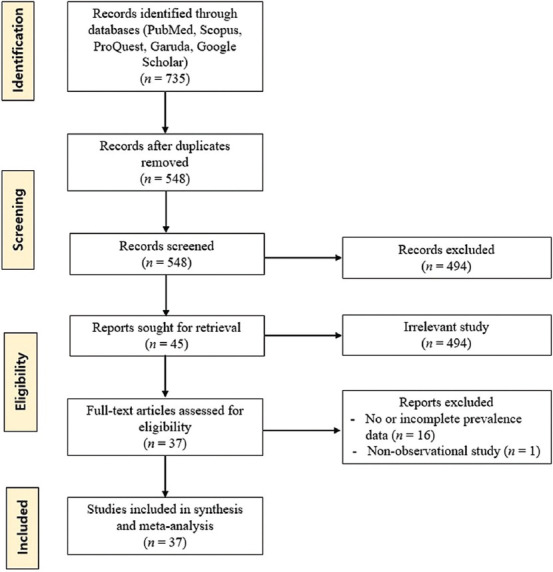
Flow diagram of eligible studies

The literature search and analysis were conducted from December 2022 to January 2023. The study was conducted using selected articles reporting dairy mastitis in Indonesia published between January 2003 and December 2022 (Figure-1). Indonesia is a tropical country located in 6° LU–11° LS and 95° BT–141° BT between the Pacific and Hindi Oceans. Indonesia has a decent livestock population, and one of them is dairy animals. The dairy industry in Indonesia is dominated by dairy cows and goats. The total dairy cattle population in Indonesia was 578,579 in 2021, of which more than 550 thousand inhabited Java Island [8]. The largest dairy cattle population can be found in East Java, with approximately 301,780 individuals. Similarly, in 2021, the goat population was also high in Java Island, with approximately 6.3 million goats or one-third of the total population in Indonesia [9]. However, the exact number of dairy goats is still unknown, but the milk production of goats was higher than that of cattle (403,174 vs. 233,923 L) in 2021, indicating a decent dairy goat population [10]. Buffalo population in Indonesia is up to 1.19 million, mostly found in Sulawesi, Kalimantan, and Nusa Tenggara [11]. However, most buffaloes were subjected to meat consumption and very rarely to milk production.

### Literature search

Before the study, a protocol was prepared as per the guideline of Open Science Framework (OSF) (https://osf.io/u9egh/) and Systematic Reviews for Animals and Food (SYREAF) (https://syreaf.org/protocols/). PubMed, Scopus, ProQuest, Google Scholar, and Garuda were systematically searched for the literature using medical subject headings terms “mastitis,” “subclinical mastitis,” “clinical mastitis,” “dairy,” “cattle,” “goat,” “buffalo,” “ruminant,” and “Indonesia” that were combined or separated using “AND”/”OR.” The selection was performed in accordance with the Preferred Reporting Items for Systematic Reviews and Meta-analysis Guidelines [12]. Figure-1 presents the flowchart showing the number of mastitis prevalence studies that were retrieved, reviewed, and collated for the meta-analysis of the prevalence of mastitis in dairy animals in Indonesia.

### Selection criteria

The selection of articles for the meta-analysis was based on the following criteria: (1) cross-sectional study; (2) peer-reviewed original articles or conference proceedings; (3) clearly stating the prevalence of dairy cattle, goat, or buffalo mastitis; (4) having a minimum of 30 samples; (5) published in English or Bahasa Indonesia; and (6) providing data of mastitis in dairy animals in Indonesia. Reports regarding the investigation of the antimicrobial resistance of mastitis-causing bacteria, knowledge of farmers on mastitis, duplicate publication or extension of the analysis from the original studies, and incomplete studies were excluded from the review process. The screening of the title and abstract in Rayyan–Intelligent Systematic Review (https://www.rayyan.ai/) included 45 reports published from 2003 to 2022 in the second eligibility screening using full text articles. A total of 37 studies met the inclusion criteria after the second screening. Most of the studies (n = 23) provide the prevalence of an animal basis, whereas the others (n = 14) present a quarter-basis.

### Data extraction

Conflicts in the screening process were solved through discussion between the two reviewers until reaching an agreement. The extracted data consisted of author name, year, location of the study, detection method, animal type, sample size, and total prevalence of mastitis (either CM, SCM, or both), and all data were collated into predesigned Microsoft Excel sheets (Microsoft Corp, Redmond, WA, USA).

### Quality assessment of individual studies

Study quality was evaluated using a checklist with a scoring based on a simple scale system following Ding *et al*. [13]. The risk of bias was determined by answering and scoring the answers of these questions: (1) Does the primary study report the prevalence of mastitis in dairy animals in Indonesia? (2) Do the title and abstract contain the population (dairy, cattle, goat, buffalo, Indonesia) and outcome (mastitis prevalence, mastitis detection)? (3) Is the full text available in English or Indonesian language? (4) Is the article original with a relevant study design? (5) Is the detection method well defined? The answers were in between yes, no, and unsure, and the scoring was 2 for yes, 1 for unsure, and 0 for no answer so the score range was 0–10.

### Statistical analysis for the overall pooled estimate of mastitis prevalence in dairy animals in Indonesia

The meta-analysis was conducted using the R software version 4.2.2 (Comprehensive R Archive Network, Vienna, Austria) using the “Meta” and “Metaphor” R packages [14]. The obtained results were represented by forest plot graphics known as confidence interval (CI) plots that display prevalence estimation and CI for each study. The estimated prevalence was indicated by the square, and the extending horizontal line from the square indicates the 95% CI. If p-value was significant, the random-effects model values in the forest plot were used to determine the estimated prevalence and CI. The estimated prevalence was expressed in percentages, along with CI and prediction interval at the 95% level.

### Subgroup meta-analysis

Subgroup analysis was conducted by identifying the heterogeneity of mastitis prevalence in four predefined groups: Province, mastitis type (SCM or CM), animal type (cow or goat), and diagnostic method. For the diagnostic method subgroup meta-analysis, only the California mastitis test (CMT) and the Institut Pertanian Bogor 1 (IPB-1) test were included in the analysis. Due to their high sensitivity and specificity, these are the most commonly used methods in mastitis detection [15, 16]. If the number of studies for certain subgroups was less than three, the subgroup was excluded from the analysis.

### Meta-regression

Meta-regression was conducted to assess the secular trend in the publication year of the study of mastitis prevalence in Indonesia following Loiklung *et al*. [17]. The publication year was used in the meta-regression assessment.

### Sensitivity analysis

The robustness of the pooled estimate of mastitis prevalence was verified through mixed-model calculations to compare the result of a fixed-effect model with that of a random-effects model (a model of choice). Leave-one-out meta-analysis was also conducted to determine if each study disproportionately influenced the study results.

### Risk of publication bias

The risk of publication bias was assessed using Egger’s test with p < 0.1 as an indicator of publication bias [18]. A funnel plot was used to visualize the publication bias assessment.

## Results

### Literature search

A total of 735 studies were retrieved and subjected to the screening process, including 37 studies with 6050 samples, ranging from 30 to 592 in the selected articles. Figure-1 presents the selection process, and Table-1 shows the characteristics of the included studies [19–55]. Based on the province, almost all of the studies were conducted in Java Island (East Java, n = 22; West Java, n = 5; Central Java, n = 5; Yogyakarta, n = 4), and two were conducted in Riau and North Sumatera. Most studies reported SCM (n = 37), and only six reported CM. The studied animals consisted of cows (n = 28), goats (n = 8), and buffalo (n = 1), which were mostly tested using CMT (n = 31) and a few of them using the IPB-1 test (n = 6).

**Table-1:** Details of dairy mastitis studies in Indonesia included for meta-analysis.

Authors and publication years	Province	Mastitis type	Animal	Diagnostic Method	No. of positive	Sample size	Prevalence (%)
Afrilia *et al*. 2021 [19]	East Java	SCM	Cow	CMT	104	136	76.47
Anggraeni and Nurfuadi, 2021 [20]	West Java	SCM	Cow	CMT	104	126	82.54
Artdita *et al*. 2020 [21]	Yogyakarta	SCM	Goat	CMT	21	204	10.29
Effendi, 2008 [22]	East Java	SCM, CM	Cow	CMT	252	308	81.82
Effendi *et al*. 2018 [23]	East Java	SCM	Cow	CMT	131	173	75.72
Effendi *et al*. 2019 [24]	East Java	SCM	Cow	CMT	128	150	85.33
Fatmawati *et al*. 2019 [25]	East Java	SCM	Cow	CMT	75	412	18.20
Fatonah *et al*. 2020 [26]	Central Java	SCM	Cow	CMT	84	120	70.00
Harjanti and Sambodho, 2020 [27]	Central Java	SCM, CM	Cow	CMT	296	412	71.84
Hasbullah *et al*. 2022 [28]	East Java	SCM	Cow	CMT	25	39	64.10
Indarwati *et al*. 2015 [29]	East Java	SCM	Goat	CMT	23	51	45.10
Khasanah and Widianingrum, 2021 [30]	East Java	SCM	Cow	CMT	39	49	79.59
Khasanah *et al*. 2021 [31]	East Java	SCM	Cow	CMT	101	148	68.24
Lidiyawati *et al*. 2020 [32]	East Java	SCM	Cow	CMT	57	136	41.91
Mardian *et al*. 2020 [33]	East Java	SCM	Goat	CMT	41	70	58.57
Nianto *et al*. 2019 [34]	East Java	SCM, CM	Cow	CMT	31	64	48.44
Nisa *et al*. 2020 [35]	East Java	SCM, CM	Cow	CMT	87	100	87.00
Permatasari *et al*. 2022 [36]	East Java	SCM	Cow	CMT	89	112	79.46
Qolbaini *et al*. 2014 [37]	West Java	SCM	Cow	CMT	86	102	84.31
Setianingrum *et al*. 2019 [38]	East Java	SCM	Cow	CMT	10	47	21.28
Setiawan *et al*. 2013 [39]	West Java	SCM	Goat	IPB-1	16	77	20.78
Sevitasari *et al*. 2019 [40]	East Java	SCM	Goat	CMT	48	58	82.76
Siagian and Amidjaya, 2022 [41]	West Java	SCM	Cow	IPB-1	29	43	67.44
Sudarwanto *et al*. 2016 [42]	North Sumatera	SCM	Buffalo	IPB-1	27	42	64.29
Sugiri and Anri, 2010 [43]	Central and West Java	SCM	Cow	CMT	325	382	85.08
Surjowardojo *et al*. 2008 [44]	East Java	SCM	Cow	CMT	21	35	60.00
Susanty *et al*. 2017 [45]	West Java	SCM	Cow	IPB-1	223	331	67.37
Sutarti *et al*. 2003 [46]	Central Java	SCM	Cow	CMT	85	237	35.86
Suwito *et al*. 2019 [47]	Yogyakarta	SCM	Goat	CMT	112	384	29.17
Suwito *et al*. 2021 [48]	Yogyakarta	SCM	Goat	CMT	54	91	59.34
Ulfaturrohmah and Surjowardojo, 2018 [49]	East Java	SCM	Cow	CMT	42	144	29.17
Utami *et al*. 2014 [50]	East Java	SCM	Cow	CMT	33	79	41.77
Wicaksono and Sudarwanto, 2016 [51]	Central Java	SCM	Cow	IPB-1	85	130	65.38
Widianingrum *et al*. 2022 [52]	East Java	SCM	Cow	CMT	397	592	67.06
Winarso, 2008 [53]	East Java	SCM	Cow	IPB-1	83	160	51.88
Windria *et al*. 2016 [54]	Yogyakarta and Riau	SCM, CM	Goat	CMT	52	93	55.91
Zalizar *et al*. 2018 [55]	East Java	SCM, CM	Cow	CMT	137	213	64.32

SCM=Subclinical mastitis, CM=Clinical mastitis, CMT=California mastitis test, IPB-1=Institut Pertanian Bogor 1

### Study quality assessment

The scoring rate of the study quality assessment is presented in Table-2. The overall quality score was 9.59 ± 0.82, whereas the median score was 10 (range, 7–10).

**Table-2:** Study quality assessment presenting the number of studies in each question.

Question	No. of included study in each category		

	Yes	No	Unsure
1. Does the primary study report the prevalence of mastitis in dairy animal in Indonesia?	30	0	7
2. Do the title and abstract contain the PO used in current study?	32	4	1
3. Is the full text available in English or Indonesian?	37	0	0
4. Is the article an original article with relevant study design?	36	0	1
5. Is the detection method well defined?	37	0	0

### Overall pooled mastitis prevalence in dairy animals in Indonesia

**Figure-2:**
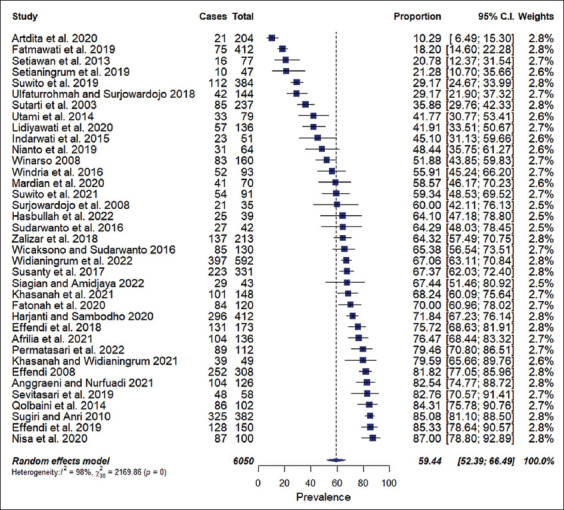
Forest plot showing pooled prevalence estimates of dairy mastitis in Indonesia

This study systematically reviewed mastitis prevalence in dairy cattle, goats, and buffalo in Indonesia. The pooled estimate of mastitis prevalence in dairy animals was 59.44% (95% CI, 52.39%–66.49%). This study found a significant heterogeneity (Q = 2169.86, df = 36, p < 0.01) between 37 studies. The heterogeneity (I^2^) index value was 98%. The forest plot (Figure-2) presents the proportion of animals affected by mastitis per study and the pooled estimate of mastitis prevalence in dairy animals in Indonesia.

### Subgroup meta-analysis

**Figure-3:**
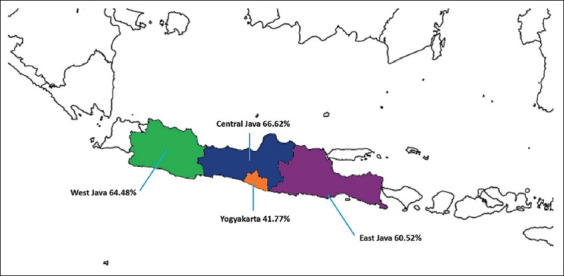
The prevalence estimates of dairy mastitis in four provinces in Java Island, Indonesia

In the province subgroup meta-analysis, two provinces in Sumatera Island, North Sumatera (64.28%) and Riau (26.67%), were excluded due to the limited number of studies; thus, only data from four provinces, all in Java Island, were analyzed. The prevalence of SCM was significantly higher than that of CM (58.24%, [95% CI, 51.26%–65.23%] vs. 3.31% [95% CI, 1.42%–5.19%]; p < 0.01) (Table-3). The prevalence of mastitis in dairy animals in four provinces was not significantly different (p = 0.47). Yogyakarta had the lowest prevalence (41.77% [95% CI, 14.96%–68.58%]), whereas Central Java had the highest prevalence (66.62% [95% CI, 49.37%–83.87%]) (Figure-3). In the animal species analysis, a study on buffalo mastitis was excluded from the study; thus, the remaining species were cow and goat. The results indicated that cows (63.42% [95% CI, 55.97%–70.86%]) had a higher prevalence than goats (44.96, [95% CI, 28.26%–61.66%], but the difference was not significant (p = 0.05). Regarding the diagnostic method, the result indicated no difference in the CMT and IPB-1 test with a prevalence rate of 60.08% (95% CI, 52.11%–68.06%) versus 56.00% (95% CI, 41.20%–70.81%), respectively (p = 0.63).

**Table-3:** Overall pooled prevalence of mastitis in dairy animal and subgroup meta-analysis using a random effect model.

Categories	No. of studies or subgroups	Prevalence		Heterogeneity			p-value for subgroup difference
	
		Estimates	(95% CI)	Q	p-value	I^2^	
Overall	37	59.44	(52.39; 66.49)	2169.86	<0.01	98	
Subgroup analysis							
Mastitis type							
Subclinical	37	58.24	(51.26; 65.23)	2075.27	<0.01	98	<0.01
Clinical	6	3.31	(1.42; 5.19)	14.81	0.01	66	
Province^a^							
Central Java	5	66.62	(49.37; 83.87)	158.18	<0.01	97	0.47
East Java	22	60.52	(51.73; 69.32)	994.99	<0.01	98	
West Java	6	64.48	(46.11; 82.86)	145.79	<0.01	97	
Yogyakarta	4	41.77	(14.96; 68.58)	155.57	<0.01	98	
Animal^b^							
Cow	28	63.42	(55.97; 70.86)	1285.55	<0.01	98	0.05
Goat	8	44.96	(28.26; 61.66)	288.79	<0.01	98	
Diagnostic method							
CMT	31	60.08	(52.11; 68.06)	2083.62	<0.01	99	0.63
IPB-1	6	56.00	(41.20; 70.81)	86.03	<0.01	94	

a Two provinces of Riau and North Sumatera were excluded from the analysis due to lack of data.

b A study about buffalo mastitis was excluded. CI=Confidence interval, CMT=California mastitis test, IPB-1=Institut Pertanian Bogor 1

### Meta-regression

Based on the meta-regression analysis, the mastitis prevalence was not significantly different in terms of the publication year (p = 0.48), with a positive increase trend of the equation: Y = −10.39 + 0.005*Year. It revealed that publications between 2003 and 2022 had similar prevalence.

### Sensitivity analysis

The calculation using the fixed-effect and random-effects models showed similar results (58.73% [95% CI, 57.66%–59.79%] vs. 59.44% [95% CI, 52.39%–66.49%]). As the results indicated the robustness of both models, we chose the random-effects to be used in our study. The results of the leave-one-out meta-analysis indicated slight changes in the overall pooled estimate of mastitis prevalence in Indonesia. The prevalence was the lowest at 58.65% (95% CI, 50.78%–67.02%) if the study by Nisa *et al*. [35] was removed and the highest at 60.85% (95% CI, 53.81%–68.80%) if the study by Artdita *et al*. [21] was excluded from the study.

### Risk of publication bias

The result of Egger’s test showed p = 0.74, which does not indicate severe publication bias.

## Discussion

The results of this study provide baseline information on mastitis prevalence in dairy animals in Indonesia. The pooled estimate of mastitis prevalence was 59.44% (95% CI, 52.39%–66.49%), dominated by reports from Java Island with more SCM than CM cases. Our pooled estimate of prevalence was higher than that in China (37.7%) [53] and Ethiopia (43.6%–47.0%) [1, 56]. In our data, SCM cases were reported in all studies (n = 37),whereas CM was reported in only six studies among all sources. Accordingly, the SCM prevalence in dairy animals was significantly higher than the CM prevalence in Indonesia, which is similar to the global trend [55]. However, the prevalence of SCM in Indonesia (58.24%) was higher than that observed worldwide (42%), in India (45%), in Ethiopia (32.21%), and in China (37.7%) [1, 3, 57, 58]. Meanwhile, the CM prevalence in Indonesia (3.31%) was lower than that observed worldwide (15%), in India (18%), and in Ethiopia (12.59%) [1, 57, 58].

The high prevalence of SCM in Indonesia indicates that more than half of dairy milk distributed in Indonesia contains high SCC or microorganisms that could alter milk quality. This could be attributed to intrinsic (age, lactation stage, breed, and parity) or extrinsic (pathogen, farm management, hygiene and sanitation, and milking procedures) factors [59]. In Indonesia, mastitis cases are associated with a lactation stage longer than 2 months, poor house cleanliness, and poor milking procedures [30, 31]. Most dairy farmers perform hand-milking procedures often without washing, pre-dipping, or post-dipping treatment [30]. The combination of an unhygienic environment and milking procedures increases the transmission of microorganisms in the udder and environment to the teat canal, eventually causing SCM [60]. Furthermore, SCM infection usually remains untreated in animals because farmers could not visually detect it [57]. Contrarily, CM is easily detected through visual changes; thus, farmers can treat the infected teat or udder quickly after noticing the infection, resulting in low CM cases [3]. Therefore, SCM infection is more common than CM infection.

In our analysis, most of the studies of mastitis in dairy animals were conducted in four provinces on Java Island, where 97% of dairy farms are located [61]. Among the four provinces (Central Java, West Java, East Java, and Yogyakarta), the pooled estimate of mastitis prevalence was not significantly different. Central Java had the highest prevalence, whereas Yogyakarta had the lowest. The similarity of mastitis prevalence in these four provinces may be associated with the similarity in the cow breed, agroclimate, and farm management. Most cow dairy farms in Indonesia are managed by small-scale farmers with less than five Friesian-Holstein cows per farm [62]. For dairy goats, the common breeds are Ettawa crossbreed, Saanen, and Sapera (Saanen and Ettawa crossbreed), with the population of dairy goats in a farm ranging from 20 to >100 [63]. However, farm management of dairy cows and goats is generally similar. The common farming management practiced in Java Island consists of feeding, gathering grass, shed cleaning, bathing cows/goats, and milking, which consume 4.4–7 h/day [64, 65]. However, the farm management of both dairy cows and goats still lacks the implementation of hygiene practices that result in a high prevalence of mastitis [30, 66].

Although the farm management of dairy cows and goats is similar, cows had a higher mastitis prevalence (66.42%) than goats (44.96%). The difference was not statistically significant (p = 0.05). The insignificant difference in statistics could be altered by the small number of study on goats compared with cows (n = 8 vs. n = 28, respectively), increasing the standard error in the study on goats. If more studies of goat mastitis are included, their difference will become significant. A similar pattern has been reported in the study by Hasan [67], which showed that mastitis prevalence was higher in cows than in goats (43% and 31%, respectively). Compared with other studies, the prevalence of dairy cow mastitis in Indonesia was higher than that reported in other countries such as Bangladesh (43%), India (39%), and Ethiopia (43%) [56, 57, 67]. Similarly, the prevalence of dairy goat mastitis was higher in Indonesia than in Algeria (8.99%), Bangladesh (31%), India (19.89%), and Brazil (30.8%) but similar to that in China (45.82%) [67–71]. The milk of cows and goats has different characteristics, including normal SCCs. In goats, the SCCs are higher, which is predominantly caused by neutrophils of up to 74%, and it plays an important role in the early defense mechanism from pathogens [72]. Meanwhile, the neutrophil content in the SCC of cow milk is about 5%–20%; thus, goat is suggested to have better resistance to natural infection than cow.

In Indonesia, mastitis in dairy animals is commonly detected using the CMT and IPB-1 test, which provide easy and fast results. Based on these methods, mastitis prevalence was not significantly different. This implies that both methods equally provide a good representation of mastitis condition. The CMT and IPB-1 test have similar principles in mastitis detection; both detect SCC within the milk by implementing viscosity changes after the cell reacts with the reagent [15, 16]. Compared with other methods, such as the breed method test, the CMT and IPB-1 test are easier to use on the farm. The breed method test requires the use of more equipment and laboratory work to detect mastitis by calculating SCC under the microscope [12]. Thus, the CMT and IPB-1 test are more popular than the breed method in Indonesia. Furthermore, both methods have high specificity in detecting SCM. The IPB-1 test was developed in Indonesia in 1998 to increase the availability of mastitis test kits; it has a sensitivity of 99%, specificity of 92%, and prediction rate of 95% [15]. Similarly, CMT has been used worldwide for detecting mastitis; it has good specificity and sensitivity even in early lactation (80.6% and 82.4%, respectively) [16]. Implementing the CMT and IPB-1 test is slightly different between dairy cows and goats. A weak positive from CMT and IPB-1 test on cattle milk is defined as a real positive case, but in goat milk, a weak positive does not account for real positive mastitis because normal goat’s milk contains high SCC that causes weak positive in both test [72]. Based on meta-regression, the pattern of mastitis cases over the period of 2003–2022 showed no significant difference. This indicates that mastitis prevalence is relatively stable over decades and that improvement is needed. Indonesian farmers have not fully implemented good dairy farming practices (GDFP) and good handling practices, particularly in the health aspect [73, 74]. Furthermore, the housing and tools aspect had a low GDFP score [74], which indicates that the farmers in Indonesia have low concern in this area. Dry cow therapy has been suggested to help reduce mastitis cases and provide protection during early lactation [73, 75]. However, this method has not been widely implemented in Indonesia. Farmers only treat animals if CM is present during the lactation period. To reduce the mastitis prevalence, the farmers’ capacity to practice hygienic farming management should be improved and a national mastitis control strategy should be developed. Reducing mastitis prevalence should increase production and enhance milk quality in Indonesia.

This study has some limitations. First, most of the studies were conducted in only four of the 36 provinces in Indonesia. Although there were reports from other provinces, two provinces in Sumatra Island were excluded from the subgroup province meta-analysis due to the limited number of mastitis studies. Considering these four provinces represent >90% of the dairy population, our result may represent the national condition. The results of this study should be used to improve dairy farm management practices and develop a mastitis control strategy to eventually alleviate the economic loss. However, only dairy cows and goats were included in the animal species subgroup analysis, as studies on dairy buffalo were lacking. Furthermore, we only used the CMT and IPB-1 test for the diagnosis because they are the most common methods for indirectly detecting mastitis. Other diagnostic methods, such as mastitis paper, detergent, and breed tests were excluded due to lack of reports.

## Conclusion

This study shows the pooled estimate of mastitis prevalence in dairy animals in Indonesia. Based on the subgroup analysis, SCM had a higher prevalence than CM, but the prevalence based on provinces, animal species, and detection methods is not different. The prevalence had a similar trend from 2003 to 2022. A mastitis control strategy needs to be developed to reduce mastitis prevalence and further loss in production.

## Authors’ Contributions

All authors participated in the study design. DMN and MA: Collected, selected, and analyzed the articles. PS: Rechecked the meta-analysis and result. DMN, MA, PS, and PP: Drafted the manuscript. All authors have read, reviewed, and approved the final manuscript.
